# Quantitative Analysis of Stress–Stretch Curves in Canine Lumbar Vertebrae Using Modified Logistic Functions

**DOI:** 10.3390/bioengineering11050516

**Published:** 2024-05-20

**Authors:** Ernest Kostenko, Rimantas Stonkus, Jakov Šengaut, Nikolaj Višniakov, Algirdas Maknickas

**Affiliations:** 1 Department of Veterinary, Faculty of Agrotechnologies, Vilniaus Kolegija/Higher Education Institution, 08105 Vilnius, Lithuania; 2 Department of Mechatronics, Robotics and Digital Manufacturing, Vilnius Gediminas Technical University, 10105 Vilnius, Lithuania; 3 Jakov’s Veterinary Centre, 03147 Vilnius, Lithuania; 4 Institute of Mechanical Science, Vilnius Gediminas Technical University, 10105 Vilnius, Lithuania; 5 Department of Biomechanical Engineering, Vilnius Gediminas Technical University, 10105 Vilnius, Lithuania

**Keywords:** canine, logistic function, lumbar vertebrae, stress, stretch

## Abstract

Background: The mechanical characteristics of bone are crucial for comprehending its functionality and response to different load conditions, which are essential for advancing medical treatments, implants, and prosthetics. By employing mathematical modeling to analyze the mechanical properties of bone, we can assess stress and deformation under both normal and abnormal conditions. This analysis offers valuable perspectives on potential fracture risks, the effects of diseases, and the effectiveness of various treatments. Therefore, researchers are attempting to find an adequate mathematical description of the mechanical properties of bone. Methods: Experimental stress–stretch external loading curves were obtained through investigations of canine vertebrae. The obtained experimental curves were fitted using the SciPy Python library with a slightly modified logistic function (logistic function plus additional const). Results: The resulting coefficient of determination $R^{2}$ (R squared) for most curves was near 0.999, indicating that an appropriate fitting function was selected for the description of the experimental stress–stretch curves. Conclusions: The stress–stretch behavior of canine vertebrae can be described using a logistic function modified by adding additional parameters for the most accurate fitting results.

## 1. Introduction

The mechanical properties of bone are fundamental to understanding its function and behavior under various loading conditions, as well as to the development of medical treatments, implants, and prosthetics. Mathematical modeling of the mechanical properties of bone allows for the analysis of stress and stretch under physiological and pathological conditions, providing insights into fracture risks, disease impacts, and treatment outcomes. Previous research has explored various mathematical models that describe the stress–stretch behavior of bone, each catering to different aspects of bone mechanics and applicable to a range of scenarios from everyday activities to traumatic impacts. Depending on the error tolerance of the model considered, it can be useful to differentiate between linear and non-linear mathematical models of mechanical stress–strain (or stretch for large deformation) of bone, including isotropic or orthotropic elastic, elastic–plastic, viscoelastic, and poroelastic models [1,2,3,4,5,6,7,8].

For non-linear modeling of biological tissues, particularly when considering large deformations that exceed the limits of traditional linear elastic models, several hyperelastic models can be applied effectively. These models are capable of capturing the complex, non-linear stress–stretch behavior of bone and other biological tissues under various loading conditions. The choice of model depends on the specific characteristics of the bone tissue studied and the nature of the deformation. The neo-Hookean model [9] is often used as a first approximation in biological tissue modeling. Its simplicity makes it appropriate for initial studies, although it may not capture the full complexity of the mechanical responses of bone. The Mooney–Rivlin model extends the neo-Hookean framework by adding an additional term to better accommodate the non-linear characteristics of materials under compression and tension. This model is more flexible and can more accurately represent the mechanical behavior of soft tissues and rubber-like materials. Its ability to fit experimental data for various deformation modes makes it a valuable tool for more detailed studies of bone and other biological tissues [10]. The Ogden model is particularly useful for capturing the complex, non-linear elastic behavior of anisotropic materials, such as bone, where the response to loading can vary significantly in different directions [10]. The Arruda–Boyce model [11] offers a micro-mechanical approach to hyperelasticity based on the statistical mechanics of polymer chains. Although this model was originally developed for polymers, its principles can be applied to modeling the non-linear elastic behavior of biological tissues, including bone, especially in contexts where the microstructure of the material plays a crucial role in its mechanical response. The Yeoh model [12] starts with a simple form that accurately captures the initial stiffness and adds higher-order terms as required to account for material responses under larger stretches. This model is useful for capturing the non-linear behavior of bone, particularly when focusing on compressive loading conditions.

The weakness of the above-mentioned non-linear bone tissue models is that they overlap in the lower, middle, or upper part of the stretch range but not over the full stretch interval from one to the value at which ultimate stress occurs. This has impelled researchers to identify the best-fitting curve for an accurate description of vertebra stress–stretch curves in external load experiments using canine vertebrae. The first such attempt involved the application of seventh-order polynomials for stress–time and second-order polynomials for stretch–time relations in a parametric form of the stress–stretch curve [13]. During the investigation of soft biological tissues, such as a canine aorta, in opposite tension tests, we have found that the stress–stretch relations of the aorta can be approximated by a logistic function [14]. The aim of the present research is to propose a new non-linear model based on a logistic function with four parameters appropriate for the approximation of stress–stretch relations in canine vertebra load tests.

## 2. Materials and Methods

### 2.1. Research Objects

In this study, we utilized lumbar vertebrae samples from three spayed mongrel canines, aged eight, nine, and ten years, with weights of 28, 26, and 20 kg, respectively. The first canine had been diagnosed with a mammary tumor, and the second with a pulmonary thromboembolism. Following these diagnoses, the owners of the dogs consented to euthanasia, and all necessary consent forms were completed accordingly. The third canine exhibited a mass located on the right side of the groin region. The owners of this dog expressed a lack of interest in determining the structure and cause of this tumor and consequently made the decision to euthanize the animal.

### 2.2. Vertebrae Preparation and Processing

**Figure 1:**
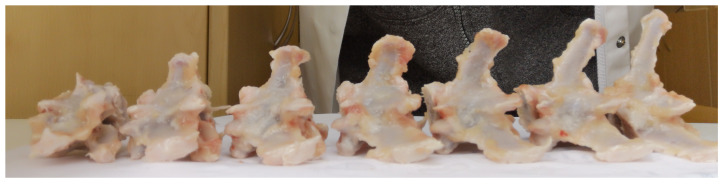
Canine vertebrae after vertebrae separation.

The animals underwent an autopsy, wherein the lumbar vertebrae from L1 to L7 were extracted, as shown in Figure 1. The dissected segment was then stored at a temperature of $-20^{\;{}^{\circ}}C$ in a freezer. For biomechanical analysis, this segment was slowly returned to room temperature ($22^{\;{}^{\circ}}C$). After thawing, the segment underwent processing, during which the surrounding muscular and adipose connective tissues were carefully removed. Each vertebra was individually isolated, and the spinal cord was excised.

### 2.3. Mechanical Experiment

Two types of equipment were used in our experiments. For load until fracture of vertebra experiments, we employed a 2055 P-5 universal tensile testing machine (Tochpribor, Ivanovo, Russia) equipped with a compression testing attachment. The canine lumbar vertebrae were subjected to vertical compressive forces through a mechanical loading system controlled by “LabVIEW version 16” (National Instruments, Austin, TX, USA). The vertebrae were aligned such that their longitudinal axes were perpendicular to the direction of the applied load, facilitating the accurate and direct force application necessary for biomechanical evaluation. Next, the compression tests utilized PXI system hardware, including the chassis NI PXIe-1073 and controller PXIe-4330 (both from Austin, TX, USA). An S-type tension/compression load cell with a maximum capacity of 1 kN was utilized. We varied the testing load velocity for different tests over the range $1-10\frac{mm}{min}$. Bone samples were frozen for storage and preservation, then thawed before testing; accordingly, our experiments used thawed wet specimens.

**Figure 2:**
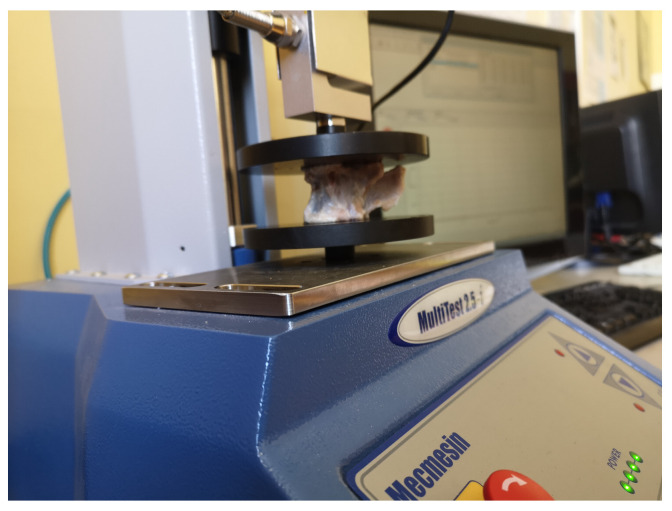
Mecmesin MultiTest 2.5-i micro-compression machine.

For the second group of tests, lumbar vertebrae were examined using a Mecmesin MultiTest 2.5-i micro-compression machine (Mecmesin Limited, Slinfold, UK) (Figure 2). The Mecmesin AFG25 cell, which controls deformation, achieved a measurement precision of within ±0.01 mm. It applied compressive forces ranging from 2 N to 2500 N with a precision of ±0.1% and maintained a crosshead speed with the same level of accuracy. During this process, each vertebra underwent compression at a force of 950 N and at a rate of 1 mm per minute. The compression process was repeated ten times for each vertebra. Post-experimentation, the axes of each vertebra were measured, and cyclic loading was applied when the specimen was in place.

### 2.4. Error Estimation

The coefficient of determination, often denoted as $R^{2}$, is a statistical measure used in the context of statistical models. Its main purpose is to predict future outcomes or test hypotheses based on related information. The coefficient of determination, $R^{2}$, is defined as follows:

(1) $$R^{2}=1-\frac{SS_{\mathrm{res}}}{SS_{\mathrm{tot}}}$$

where $SS_{\mathrm{res}}$ is the sum of squares of residuals, also known as the residual sum of squares:

(2) $$SS_{\mathrm{res}}=\sum_{i=1}^{n}{\left(y_{i}-{\hat{y}}_{i}\right)}^{2}$$

and $SS_{\mathrm{tot}}$ is the total sum of squares (a measure of the total variance in the observed data):

(3) $$SS_{\mathrm{tot}}=\sum_{i=1}^{n}{\left(y_{i}-\bar{y}\right)}^{2}$$

where $y_{i}$ is the observed outcome, ${\hat{y}}_{i}$ is the predicted value for the *i*-th observation, and $\bar{y}$ is the mean value of the observed data.

In practice, the coefficient of determination is used as an indicator of the goodness of fit of a model. A higher $R^{2}$ value indicates a better fit and suggests that the model can better explain the variation in the output using different independent variables. However, it should not be the sole criterion for model selection, especially because it can increase with the number of predictors without improving the predictive power of the model.

The confidence intervals for the lengths and loading surfaces of the measured vertebrae were calculated using the formula provided in reference [15], as follows:

(4) $$\delta x=\pm ts/\sqrt{n}$$

where *t* represents the Student’s t-value, *s* is the standard deviation, and *n* denotes the degrees of freedom plus one. For two degrees of freedom, the Student’s t-value is set at 4.303, corresponding to a 95% confidence interval [15]. Lastly, the error propagation for functions involving two variables is applied to the external pressure-induced stress, denoted by σ, as follows:

(5) σ=F/S,

where F is the measured load force and S is the vertebra loading surface. λ is the stretch, which is calculated as

(6) λ=(L−l)/L,

where *L* is vertebra length between loading surfaces and can be expressed as follows [16]:

(7) $$\delta \sigma /\left|\sigma \right|=\sqrt{{\left(\delta F/F\right)}^{2}+{\left(\delta S/S\right)}^{2}}$$

(8) $$\delta \lambda =\sqrt{{\left(\delta l/L\right)}^{2}}$$

### 2.5. Approximation of Stress–Stretch Curves

Parametric equations are often employed to describe the coordinates of points forming a geometric entity, such as a curve. In this context, the equations are jointly referred to as a parametric representation [17,18]. The experimental data-gathered stress $\sigma_{k}\left(t\right)$ on the increasing and decreasing external load and displacement, $\lambda_{k}\left(t\right)$, of the vertebra can be effectively represented using the following parametric formulas:

(9) $$\begin{array}{ccc} \sigma_{k}\left(t\right) & = & C_{0k}+\frac{C_{1k}}{1+\exp\left(-C_{2k}\cdot \left(t-C_{3k}\right)\right)}, \end{array}$$

(10) $$\begin{array}{ccc} \lambda_{k}\left(t\right) & = & D_{2k}\cdot t^{2}+D_{1k}\cdot t+D_{0k}, \end{array}$$

where *t* represents the independent variable of time, and $C_{ik}$ and $D_{ik}$ are constants specific to the material for each loading/unloading cycle denoted by *k*. The proposed fitting curve can be characterized by a tangent at point $t_{0}=C_{3k}$, as follows:

(11) $$\tan\left(\alpha \right)=L_{k}^{\prime }\left(t\right){|}_{t=C_{3k}}=\frac{C_{1k}\cdot C_{2k}}{4}$$

where angle α is tilt angle relative to the x-axis.

**Figure 3:**
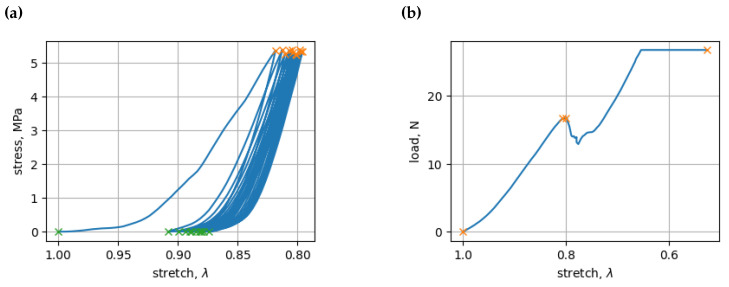
(**a**) Minima (green x) and maxima (orange x) of experimental stress values of a cyclic load; (**b**) minima and maxima of experimental stress values of ultimate load.

**Figure 4:**
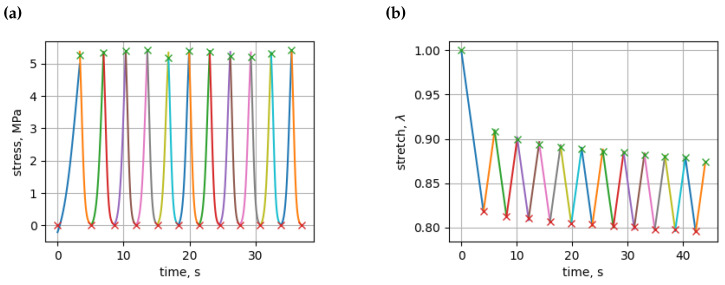
(**a**) Stress approximations by logistic function for each cycle; (**b**) stretch approximations by second-order polynomials for each cycle.

We investigated the mechanical properties of vertebrae in two types of experiments. The first type of experiment was initiated by subjecting the vertebrae to cyclic loads using a Mecmesin MultiTest 2.5-i micro-compression machine (Mecmesin Limited, Slinfold, UK), which resulted in the formation of hysteresis loops, which were observed after ten compression cycles, as shown in Figure 3a. In the second type of experiment, we observed vertebrae under a load value, eventually cracking the vertebrae using a 2055 P-5 universal tensile testing machine, as shown in Figure 3b. We then analyzed these loops to identify their minimum and maximum values. Subsequently, we extracted the peak values for stress and stretch over time. Utilizing these peaks, we were able to characterize each distinct compression–relaxation cycle, necessitating individual approximations for each (see Figure 4a,b). Subsequently, we approximated the time indexes of the peak stress and stretch values using a modified logistic function with four parameters and a polynomial with three parameters for stress and stretch, respectively. To achieve this, we employed numerical optimization using the SciPy v 1.10.1 optimize curve_fit function, as explained in [19].

In living organisms, the hysteresis loop varies because of the compensatory and decompensatory mechanisms at play. During the first cycle (as shown in Figure 3a), the vertebrae adjusted to the applied load. Clear hysteresis loops consistent with our proposed approximations were observed in the subsequent second to tenth cycles, as is described in the next section.

## 3. Results and Discussion

**Figure 5:**
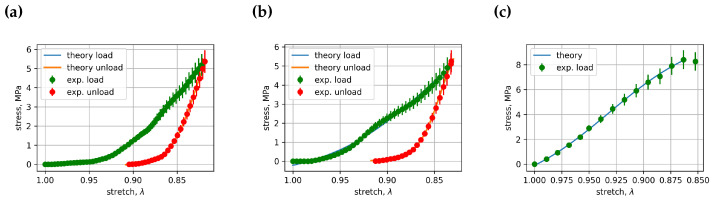
Comparison between the stress–stretch relationships for experimental and fitted curves: (**a**) cycle 1, vertebra L3, experiment 1, (**b**) cycle 1, vertebra L3, experiment 2, (**c**) cycle 1, vertebra L3, experiment 3.

**Figure 6:**
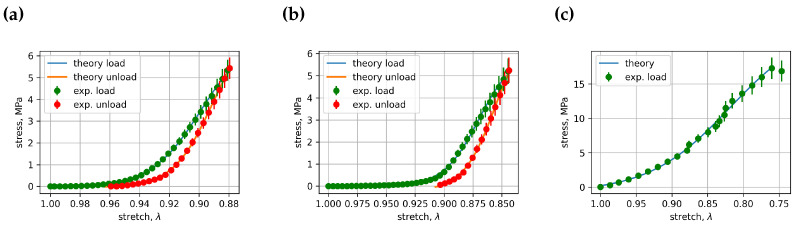
Comparison between the stress–stretch relationships for experimental and fitted curves: (**a**) cycle 1, vertebra L4, experiment 1, (**b**) cycle 1, vertebra L4, experiment 2, (**c**) cycle 1, vertebra L4, experiment 3.

**Figure 7:**
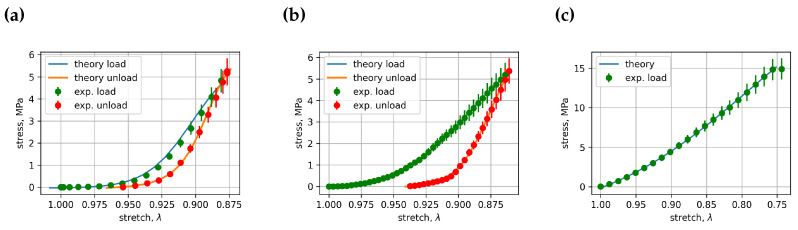
Comparison between the stress–stretch relationships for experimental and fitted curves: (**a**) cycle 1, vertebra L5, experiment 1, (**b**) cycle 1, vertebra L5, experiment 2, (**c**) cycle 1, vertebra L5, experiment 3.

All curve-fitting coefficients $C_{ik}$ and $D_{ik}$ for vertebra loading and unloading in cyclic load experiments and in vertebra load until fracture experiments are collected into the coefficients data file. This file, with 491 rows of coefficient data, can be found in the Supplementary Material [20]. All experimental and fitted stress–stretch curve images (Figures S1–S128) can be downloaded from the same open data cloud server. Some of these images, in particular Figure 5, Figure 6 and Figure 7, represent comparisons of stress–stretch relations for experimental and fitted curves. Figure 5 presents cycle 1 and vertebra L3’s loading and unloading stress–stretch relations; Figure 6 presents cycle 1 and vertebra L4’s loading and unloading stress–stretch relations; Figure 7 presents cycle 1 and vertebra L5’s loading and unloading stress–stretch relations. Examples of the fitted curves’ coefficients and their $R^{2}$’s (R squared) for the above-mentioned figures can also be found in Table 1 and Table 2. The resulting coefficient of determination for most curves was greater than 0.999, indicating that an appropriate fitting function was selected for the description of the experimental stress–stretch curves. The number of experimental cases in Table 1 and Table 2 is different because the fitting in the first two experiments was optimized for the stretch dependence time parameter *t*, whereas the third experiment included only one loading cycle, for which we used stretch as the independent parameter in the logistic curve-fitting algorithm. In general, this approach is applicable when the force cell moves at a constant velocity. For all experimentally observed cases, this is true with one exception: vertebra L5 in experiment 1.

In Table 1, the columns are as follows: the first column denotes the vertebra, which was used as the experiment name; the second column denotes the experiment number; the next four columns show the values of the fitted coefficients $C_{i}$; the $R^{2}$ column shows the ‘R squared’ values for each fitted curve; and the final column indicates the experiment type, i.e., ’load’ or ’unload’. Coefficients $C_{i}$ can be interpreted as follows: $C_{0}$ is the shifting value of the logistic function in the direction of the stress axis; $C_{1}$ is a theoretical limit of the ultimate strength; $C_{2}$ is the exponential increasing/decreasing scale ratio; and $C_{3}$ denotes the middle of the logistic curve (i.e., the maximum α value).

The $D_{1}$ coefficient values for vertebrae L3, L4, and L5 for the first loading–unloading cycle in two different experiments are presented in Table 2. All coefficient values can be found in Tables S1–S3. As shown in the results tables (Table 2 and Table S3), the stretch in most cases was linear and a function of time, with the exception of vertebra L5 in experiment 1. In all other cases, the proposed logistic function can be expressed as a logistic function of stretch with transformed linear coefficients $C_{2}^{\prime },C_{3}^{\prime }.$ as functions of $C_{i}$ and $D_{i}$.

Finally, the fitting function behavior of the system can be considered. For a sufficiently small *t*, logistic functions can be approximated as the exponential function $\sigma_{k}\left(t\right)\approx C_{0k}+C_{1k}e^{C_{2k}\left(t-C_{3k}\right)}$. This behavior exhibits good agreement with the findings of other studies concerning the stress–stretch exponential behavior of elongating stretches [21,22]. For near-maximum values of *t*, the logistic function flattens and reaches its constant threshold values $C_{0k}+C_{1k}$; in mechanical terms, this indicates that the ultimate strength of the biological material has been achieved. On the other hand, the stretch parametric curves in most measured cases were linear. Therefore, in most cases, after the rearrangement of the stretch–time relation with *t* a function of stretch and the insertion of this new function into the logistic function, we obtain a stress–stretch logistic function. This was demonstrated in the third experimental stress–stretch fitting curve, in which good R squared values were obtained.

## 4. Conclusions

Investigations of three different canine vertebrae, L1, …, L7, under an external load have allowed experimental stress–stretch curves to be obtained. The experimental stress–stretch dependence of canine vertebrae was modeled as a logistic function. To achieve better fitting results, the logistic function was modified using additional constant parameters. The resulting coefficient of determination, $R^{2}$, for almost all curves was near to or greater than 0.999, indicating that an appropriate fitting function was selected for the description of experimental stress–stretch curves. The proposed mathematical model accurately describes the investigated experimental stress–stretch dependence over the whole range of the experiment, from the beginning of loading until the ultimate strength values were reached or the vertebra fractured.

## Figures and Tables

**Table 1:** $C_{i}$ coefficient values for vertebrae L3, L4, and L5 during the first loading and unloading cycle in three different experiments.

Vertebra	Exp.	$C_{0}$	$C_{1}$	$C_{2}$	$C_{3}$	$R^{2}$	Stress
L3	1	0.1133	7.163	1.4699	3.2392	0.999	load
		−0.047	7.6214	3.1659	4.2821	0.9998	unload
	2	0.8875	10.1438	0.8824	2.9998	0.9934	load
		0.0057	6.9451	4.0148	3.6638	0.9993	unload
	3	−2.4883	13.204	22.166	0.932	0.9992	load
		-	-	-	-	-	unload
L4	1	0.0467	7.1418	26.6721	0.2254	0.9996	load
		−0.0518	7.6406	34.3704	0.2986	0.9998	unload
	2	0.0107	6.0252	3.2338	2.6063	0.9989	load
		−0.2039	7.7396	3.5797	3.4333	0.9995	unload
	3	−0.7793	24.651	17.5473	0.8189	0.9966	load
		-	-	-	-	-	unload
L5	1	0.0356	6.3102	242.6306	0.0292	0.9995	load
		−0.0566	5.7441	296.3236	0.0453	0.9994	unload
	2	0.2138	7.3233	1.794	2.1683	0.9996	load
		−0.0584	7.0522	3.827	3.1042	0.9994	unload
	3	−4.3779	33.41	9.1167	0.7886	0.9996	load
		-	-	-	-	-	unload

**Table 2:** $D_{i}$ coefficient values for vertebrae L3, L4, and L5 during the first loading and unloading cycle in two different experiments.

Vertebra	Exp.	$D_{2}$	$D_{1}$	$D_{0}$	$R^{2}$	Direction
L3	1	−0.00002	−0.4544	1.00016	0.99999	down
		0.00055	0.039999	0.64854	0.99993	up
	2	−0.00006	−0.04981	1.00012	0.99999	down
		0.00149	0.03695	0.68887	0.99941	up
L4	1	0.02194	−0.45941	1.0009	0.99988	down
		0.03257	0.43083	0.75964	0.99998	up
	2	0.00041	−0.05105	1.00035	0.99991	down
		−0.00201	0.06462	0.65712	0.99907	up
L5	1	−21.63102	−3.04609	1.00823	0.99321	down
		42.69855	−0.83142	0.84651	0.99158	up
	2	0.00013	−0.05029	1.0002	0.99999	down
		0.00051	0.04624	0.72708	0.99998	up

## Data Availability

The following experimental data files can be downloaded https://doi.org/10.18279/MIDAS.MathCurves.243873, accessed on 3 May 2024.

## Supplementary Materials

The following supporting information can be downloaded at: https://doi.org/10.18279/MIDAS.MathCurves.243873, accessed on 3 May 2024. Figures S1–S128: Comparisons of all stress–stretch relations for experimental and fitted curves; Tables S1–S3: Tables of all $C_{i}$ and $D_{i}$ coefficient values.
